# Transcriptomic profiles in peripheral blood between women with unexplained recurrent implantation failure and recurrent miscarriage and the correlation with endometrium: A pilot study

**DOI:** 10.1371/journal.pone.0189159

**Published:** 2017-12-07

**Authors:** Jin Huang, Nana Jin, Hao Qin, Xiao Shi, Yingyu Liu, Wingching Cheung, Chi Chiu Wang, Ting Fung Chan, Tin Chiu Li

**Affiliations:** 1 Department of Obstetrics and Gynaecology, the Chinese University of Hong Kong, Hong Kong SAR, China; 2 School of Life Sciences, the Chinese University of Hong Kong, Hong Kong SAR, China; 3 School of Biomedical Sciences, the Chinese University of Hong Kong, Hong Kong SAR, China; 4 Li Ka Shing Institute of Health Sciences, the Chinese University of Hong Kong, Hong Kong SAR, China; CHA University, REPUBLIC OF KOREA

## Abstract

**Aim:**

To study the transcriptome profiles in the blood of recurrent implantation failure (RIF), recurrent miscarriage (RM) and fertile women during the window of implantation, and further analysis the correlation of transcriptome profiles between blood and endometrium.

**Methods:**

This is an observational prospective study. In total 9 subjects were recruited, 3 RIF, 3 RM, and 3 controls. Paired samples (endometrium and peripheral blood) from the same subjects were precisely timed on the 7th days after luteal hormone surge (LH+7). RNA sequencing was applied to investigate the transcriptome profiles.

**Results:**

The results of transcriptome in peripheral blood cannot be used to characterize women with RIF and unexplained RM. There was a medium level correlation between transcriptome in peripheral blood and endometrium during the window of implantation.

**Conclusion:**

The differential transcriptome patterns in blood are not representative of those in endometrium, and the blood transcriptome cannot differentiate among the women with RIF, RM or fertile.

## Introduction

The endometrium becomes receptive to the embryo only in the mid-luteal phase, but not in other stages of the menstrual cycle, and it has been considered as the initial critical stage of implantation. The specific time when the endometrium becomes receptive to the embryo is often referred to as the window of implantation (WOI). Implantation failure is considered to be an important cause of infertility, whereas defective implantation may lead to miscarriage, either sporadic or recurrent [1, 2]. In clinical practice, endometrial receptivity may be investigated by morphological, immune-histochemical or genomic study of a biopsy specimen [3] but the biopsy procedure itself may disturb the implantation process and cannot be carried out, for ethical reasons, in cycles when conception may take place. Non-invasive approaches such as ultrasonographic measurement of endometrial thickness and blood flow are of limited use [4] and provide very little information on the very rapid biological changes occurring in endometrium at this specific time. To achieve a breakthrough in the understanding of the complex processes involved in human implantation requires the development of a non-invasive method which can be repeated serially but which can accurately reflect the physiological changes in the endometrium.

Peripheral blood, a heated target for liquid biopsy, contains proteins, DNAs and RNAs from various sources of cells, which could provide a special window to measure specific information for organs required monitoring [5, 6]. Transcriptomic study using micro-array or RNA sequencing enables the simultaneous study of most of the genes involved in the implantation process. Several studies have used this approach to examine the endometrium in the peri-implantation period of women with recurrent implantation failure (RIF) [7–9] or recurrent miscarriage (RM) [10, 11]. Our previous study has found different pathway regulations between RIF and RM [12]. Furthermore, whole blood transcriptome studies could reveal other physical changes of human body, like blood pressure [13], pre-term labor [14], or immune response [15]. However, none of the earlier studies applied this technique to compare and correlate the transcriptome profiles of endometrium and peripheral blood and examine the potential usefulness of transcriptome study of peripheral blood to characterize different groups of women with reproductive failure.

In this study, we wish to investigate whether there is a correlation between the transcriptome in peripheral blood and endometrium during the window of implantation, and to know whether transcriptome in peripheral blood seven days after the LH surge (LH+7) could be used to characterize women with RIF and RM.

## Materials and methods

### Subject

Three groups of subjects were recruited from the Prince of Wales Hospital, Chinese University of Hong Kong. The inclusion criteria of all subjects recruited include: age no more than 40 years, with regular cycles (25–35 day), had not used steroid hormone in the preceding 2 months. The exclusion criteria were, briefly: chromosomal anomaly, positive for anticardiolipin antibody or lupus anticoagulant, abnormal thyroid function test, uncorrected uterine anomalies, intra-uterine device in situ, or serious systematic disease. Women with unexplained RIF was defined as failure to achieve a clinical pregnancy after transfer of at least four good-quality embryos in more than three cycles with her age under 40 years, and no obvious cause had been identified [1]. Unexplained recurrent miscarriage was defined as three or more consecutive miscarriages before 24 weeks of gestation [2]. Fertile control subjects referred to women who had one or more live birth following spontaneous conception, stopped breastfeeding for more than 6 months, and without any history of spontaneous miscarriage. In total, 9 women were recruited, 3 women with unexplained RIF, 3 women with unexplained RM, and 3 fertile controls.

### Endometrial and peripheral blood samples

In the cycle of study, all subjects started daily urine LH test from day 9 of the cycle onwards until the LH surge had been identified. An endometrial biopsy was obtained on day LH+7 as an outpatient procedure with the use of a Pipelle® sampler. The samples were immediately snap-frozen and stored in liquid nitrogen for later processing. Simultaneously, 3ml of peripheral blood was collected in one dipotassium ethylenediaminetetraacetic acid (EDTA) Vacuette® tubes. Blood was mixed with TRIzol LS (Invitrogen) in a ratio of 1:3 immediately after collection, and after homogenization, the samples were stored in -80°C for later processing. The whole process blood collection, homogenization, and storage would finish within 30 minutes.

### RNA extraction and expression calculation

Total RNA was extracted from endometrium by TRIzol according to manufacturer’s protocols (Invitrogen). Blood total RNA was extracted by RNeasy Mini Kit (QIAGEN). RNA quality control was confirmed by Bioanalyzer 2100 Eukaryote Total RNA Pico (Agilent Tech, Inc). There were 9 pairs of RNA samples (3 with RIF, 3 with RM, 3 controls) with high total RNA quality and integrity for RNA-seq. All total RNA samples were rRNA depleted by Ribozero (Illumina) and the paired-ends strand-specific libraries were prepared by TrueSeq Stranded Total RNA Library Prep Kit (Illumina). All samples were sequenced by Illumina HiSeq2000. After sequencing, low quality reads whose sequencing quality below 20 were trimmed. All reads were mapped to human genome hg38 by Tophat2 [16] with default parameters. The Reads Per Kilobase Per Million Reads (RPKM) of gene expression was calculated based on the GENCODE v23 annotation [17]. All expressions were normalized by quantile normalization method using median [18].

### Unsupervised hierarchical clustering and principle component analysis (PCA)

The unsupervised hierarchical clustering and PCA were done as previously described [12]. Briefly, the raw normalized expression of genes were scaled and used for unsupervised hierarchical clustering and PCA by R packages gplots [19] and prcomp. After PCA was done, the vector of each principle component was calculated and support vector machine (SVM) was performed by Python library sklearn 0.17.0 [20]. Genes whose absolute values of the contribution scores were larger or equal to 0.01 were considered to have significant contribution.

### Gene ontology and pathway analysis

Genes with significant contribution scores were retrieved for gene ontology (GO) and pathway analysis. Pathway enrichment was analyzed by DAVID 6.7 (the Database for Annotation, Visualization and Integrated Discovery) [18].The pathways whose correlated p-value (q-value) smaller than 0.05 were considered significantly enriched [21].

### Blood and endometrium correlation analysis

Coefficients of all the genes among three groups were calculated by Pearson’s correlation. And the association of gene expressions in blood and endometrium in paired samples were also tested by Spearman’s correlation. The difference of coefficients among three groups was compared by t test.

### Ethics

This study was approved by the Joint Chinese University of Hong Kong–New Territories East Cluster Clinical Research Ethics Committee. Informed written consent was obtained from all participants.

## Results

The demographics of the recruited subjects are summarized in S1 Table. There was no significant difference in age, BMI, cycle length and endometrium thickness at the time of biopsy amongst RIF, RM and fertile groups.

### Differentially expressed genes in peripheral blood

The reads mapping of all 9 pairs of samples for RNA-Seq were satisfactory. For the blood samples, after trimming the adapters and low quality bases (Q<20 in 4bp sliding window) using Trimmomatic (v0.32), RNA-Seq reads were mapped to the human genome from the Genome Reference Consortium (GRCh38) using Tophat2 and Cufflinks. The average percentage of mapped reads was 87.38%, including 19.44% in intronic region, 37.96% in protein coding region, 0.0064% in rRNA region and 42.59% in other regions (e.g. intergenic, antisense strand etc.). Of the same data size, median expression of genes in endometrium were about 3 times higher than those in blood of each paired samples in Table 1. The raw sequencing data was uploaded to NCBI with reference BioProject ID: PRJNA379542.

**Table 1:** The median expression of genes in each paired samples.

	Endometrium (reads/gene)	Blood (reads/gene)	Ratio Endometrium/Blood
RIF1	13.4	3.4	3.9
RIF144	18.5	4.9	3.8
RIF158	14.7	5.2	2.8
RM5	14.2	5.5	2.6
RM6	12.7	5.9	2.2
RM8	13.7	5.9	2.3
C2	12.9	6.3	2.0
C3	13.5	3.1	4.4
C42	16.6	5.4	3.1

**Fig 1:**
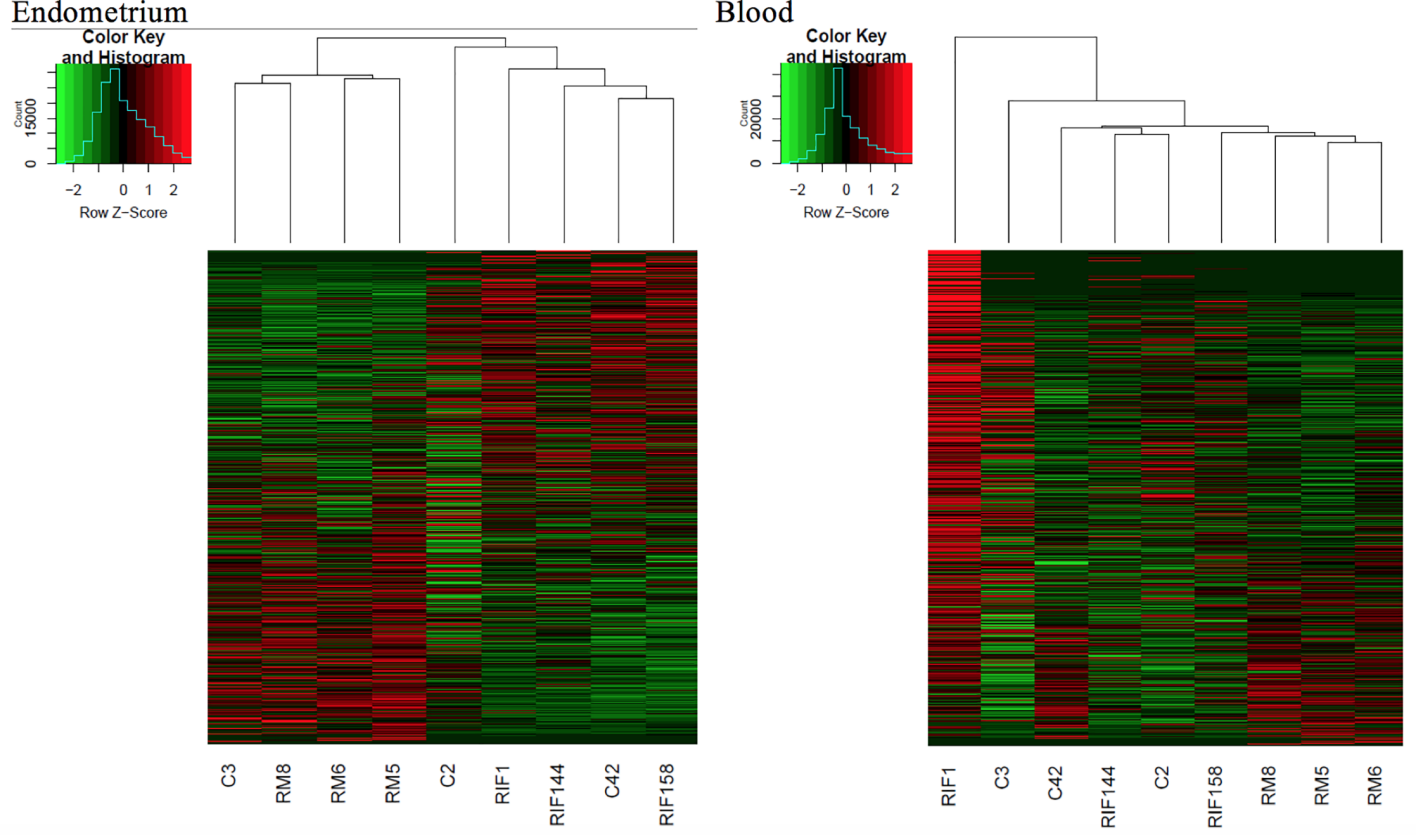
Unsupervised clustering of all gene expression in 9 paired samples, 3 with recurrent implantation failure (RIF), 3 with recurrent miscarriage (RM), and 3 fertile controls (C).

**Fig 2:**
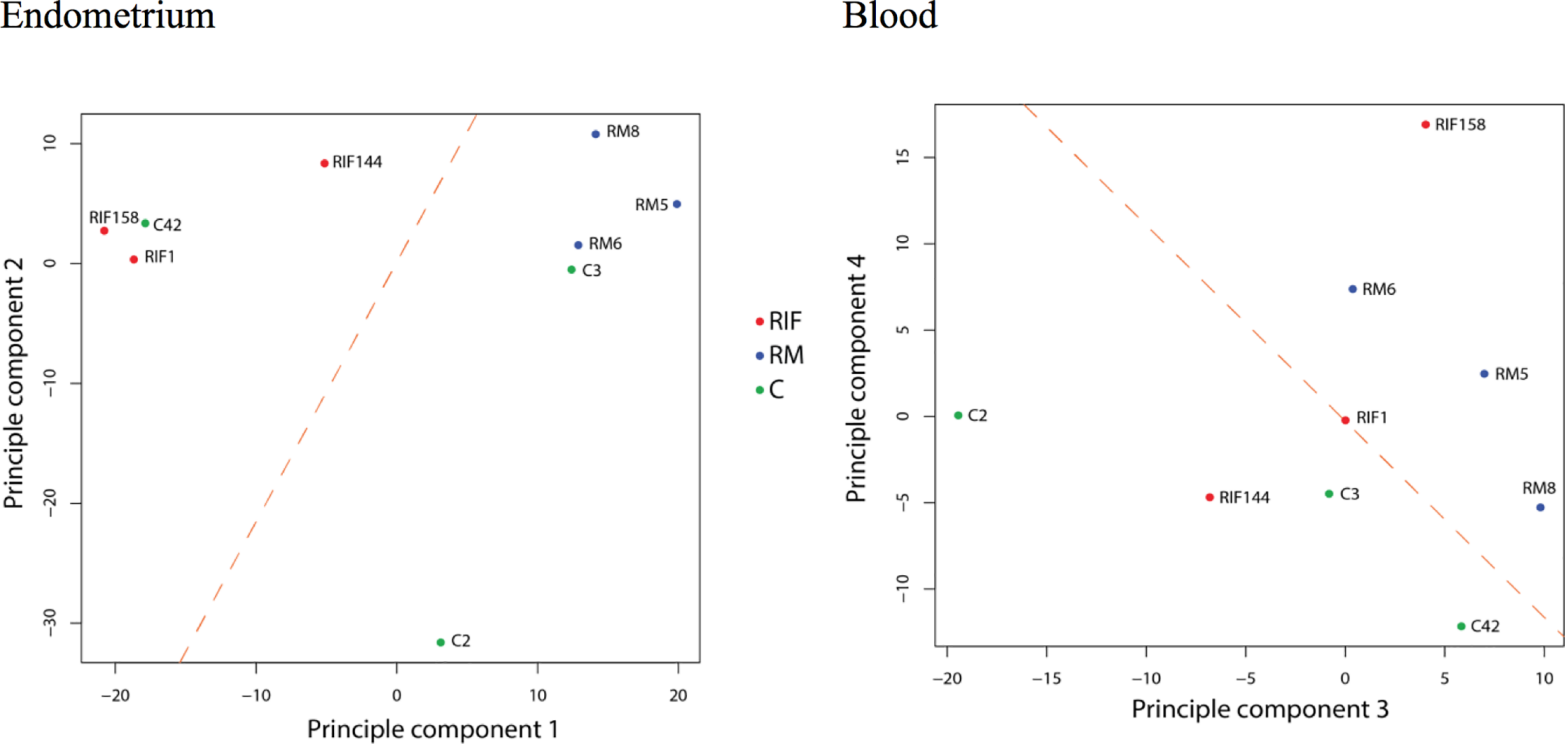
Linear separations of RIF, RM and control on principle component analysis (PCA) by support vector machine (SVM). RIF: recurrent implantation failure; RM: recurrent miscarriage; C: fertile control.

All genes were included in the unsupervised clustering, and there were 19,079 genes in endometrium and 18,446 genes in blood. Among the three studied groups, RM and RIF showed distinct different gene expression patterns in unsupervised clustering in endometrium; whereas in peripheral blood, though the 3 RM cases grouped together, the difference from the other two groups was not distinct in Fig 1. To further examine the differences between RIF and RM, principle component analysis (PCA) was performed. In endometrium, RM and RIF samples showed distinct spatial distribution in the three-dimensional space constructed by the first two components; whereas in blood, RM and RIF could not be linearly separated, and only RM and control could barely be separated in components 3 and 4 in Fig 2. Pathway analysis by DAVID 6.7 identified no significant pathways among the gene expression profiles in peripheral blood of the three groups in S2 Table.

### Correlated gene expression of peripheral blood and endometrium

**Fig 3:**
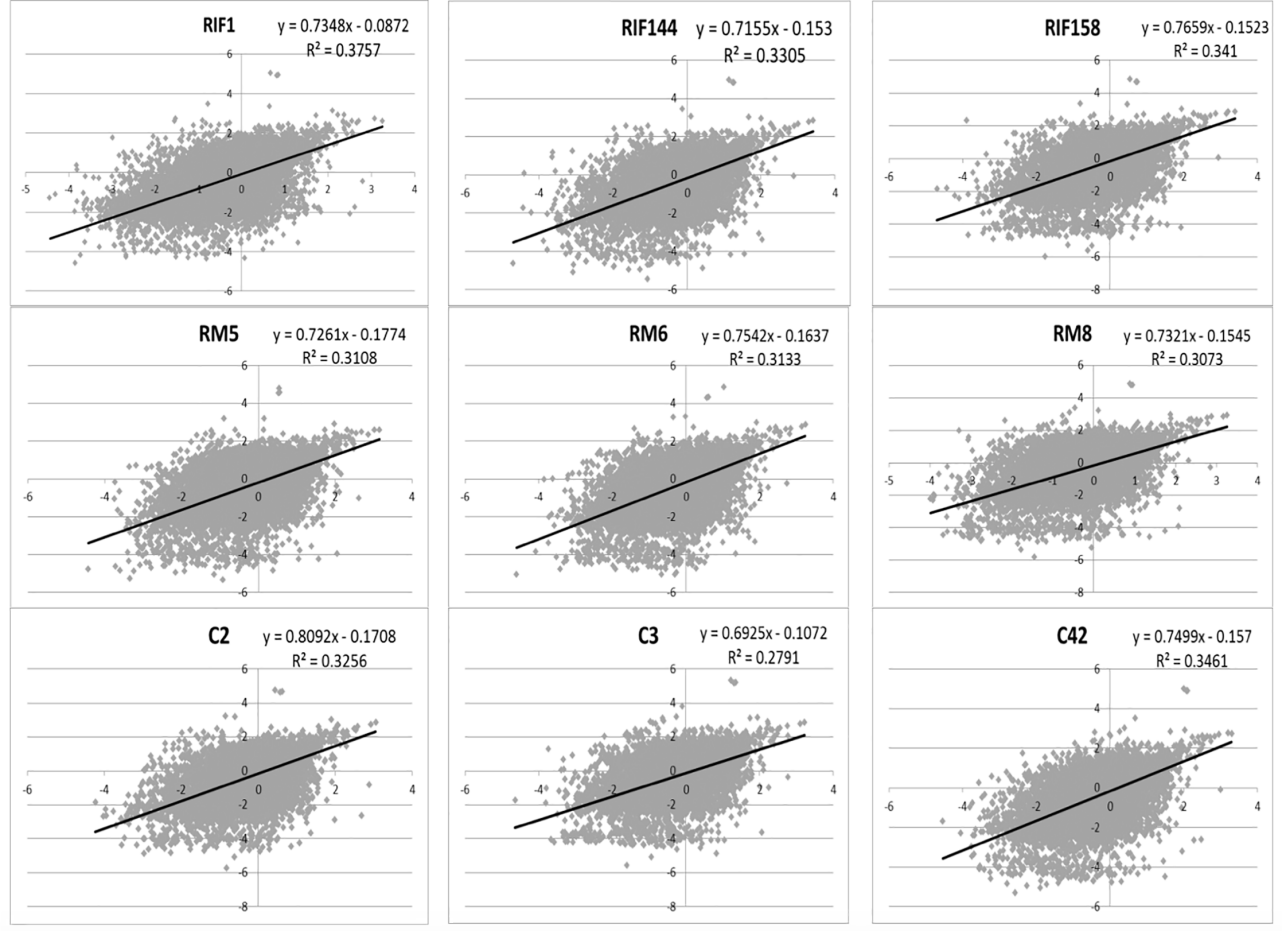
Scattered plots showed positive correlation of transcriptome in paired samples of each subjects. RIF: recurrent implantation failure; RM: recurrent miscarriage; C: fertile control.

**Fig 4:**
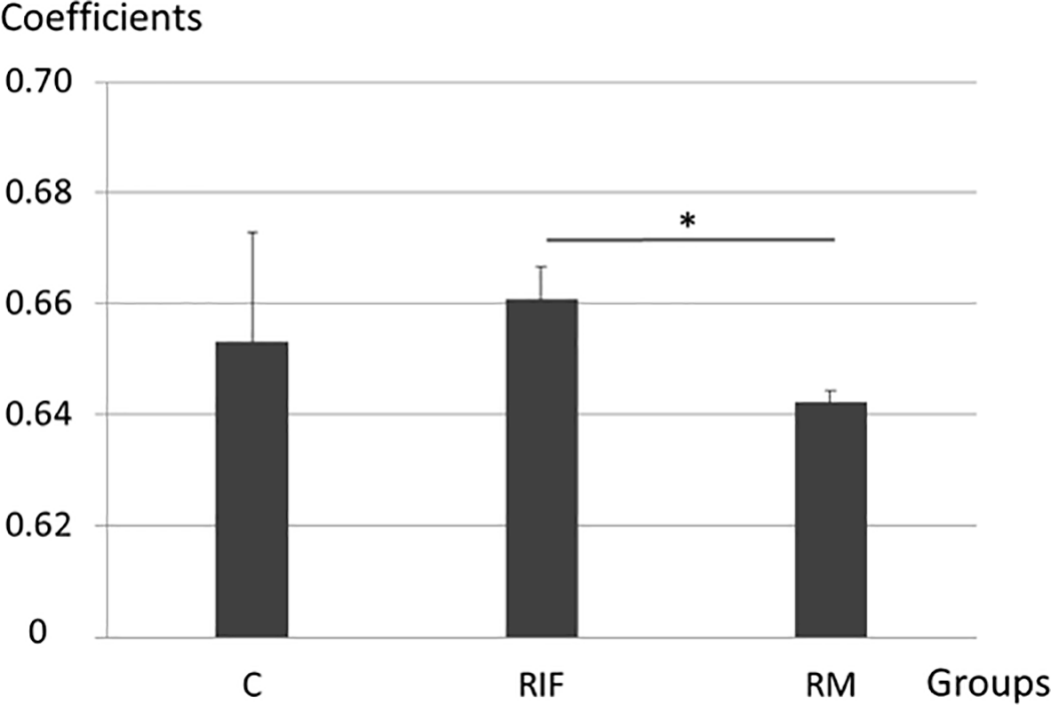
Histogram showing the coefficients of Spearman's rank correlation in different groups. Results shown were mean ± SE. *t test p = 0.027; SE: standard error; RIF: recurrent implantation failure; RM: recurrent miscarriage; C: fertile control.

Of all the genes (18446) which had sequencing reads, there were 1960 genes correlation coefficients more than 0.5, among which 126 genes involved in immune response. The correlation coefficients of the 9 paired samples were all over 0.64 and the R^2^ for each paired samples were about 0.3 in Fig 3, and it seems that the correlation between blood and endometrium were higher in RIF than those in RM, with p < 0.05 in Fig 4. As the value of correlation coefficients over 0.5 was considered as a moderate positive relationship and 0.7 as a strong positive relationship [22], the correlation of gene expression profiles between peripheral blood and endometrium were moderate.

## Discussion

In this study, we have found that the transcriptome profiles in peripheral blood of the 3 groups studied (RM, RIF and fertile controls) may not be as distinctively different from one another as in those of endometrium in Figs 1 and 2. We observed that controls C3 and C42 appeared to have rather different gene profiles. One possible explanation and a limitation of our study is the biological repeat of samples (3 in each group) is relatively small in the study human whole blood, known to contain various cell subtypes and heterogeneous molecules, which may mask the critical signature difference [23]. Therefore, no significant differentially expressed pathways could be drawn from blood samples from the 3 groups.

However, we have found correlated gene expression between blood and endometrium. Over 10.6% (1960/18446) genes in blood showed similar expression trend as in endometrium among three groups could potentially represent the changes in endometrium, in which about 6.4% (126/1960) genes involved in immune response. Tissue transcriptome correlations with peripheral blood are gradually explored in searching of potential biomarkers to investigate the functional changes of the targeted tissue. For example, circulating RNAs provide a potential approach to investigate placental transcriptome, and some RNAs presenting in maternal blood are proved to be produced by placenta [24]. Through investigating the differential transcriptome profiles in peripheral blood, several critical RNAs have been identified as biomarkers in pregnancy complications, such as preeclampsia [25–27], fetal growth restriction [28], and even real-time monitoring fetal hypoxic status [29]. It suggested that circulating RNA could be used dynamically to interrogate changes in fetal and placental health. However, so far there is no report of correlations between peripheral blood and endometrium transcriptome.

Hence, novelty of this study is for the first time trying to investigate the transcriptome in paired samples among three groups of women. Previous studies had either measured individual markers or RNA markers in blood or endometrium [30]. We have chosen in our analysis to use support vector machine to draw a two-class comparison; we acknowledge that other clustering methods could be used instead. We consider that the use of unsupervised analysis strategy helped to reduce the selection bias. Therefore, our study suggests that it is possible that more genes could be identified in blood as non-invasive markers to represent the correlated gene expression levels in the endometrium.

The relative gene expression level of whole blood to endometrium is another valuable contribution of this study for exploration of circulating markers of endometrium receptivity. As suggested by the ratio of medium expression levels of genes in each paired samples is 2–4 in Table 1, for better exploring the difference of blood transcriptome difference, about 3 times sequencing coverage should be necessary to reach the same gene expression level as that of endometrium. This could be of special importance when performing investigations for those low-level expressed correlated genes in both endometrium and blood.

Previous studies had successfully used transcriptomic profiles of the endometrium to characterize different groups of women with reproductive failure (RIF [7–9, 31, 32] or RM [10–12]); our observation suggested that it was not possible to do so with transcriptomic profile of the peripheral blood. It raises questions about the possible clinical usefulness of the peripheral blood to monitor the molecular changes in the endometrium in the window of implantation. Correlations of the transcriptome profiles are quite good between endometrium and peripheral blood given that no gene selection is being made. The difference in the genes expressed among the three groups of women is not consistently observed, but rather a relevant subset related to the condition of interest. To further explore the gene expression features of different groups, more samples analyzed with another selective strategy may be need.

Whilst transcriptomic profiles of the peripheral blood do seem to have a moderate degree of correlation with profiles of the endometrium around the time of implantation, this indirect & non-invasive approach does not seem able to replace the direct but somewhat invasive approach (endometrial biopsy) to characterize different groups of women with reproductive failure. Consequently, it is still not yet possible to use serial (for example daily) transcriptomic profiling of the peripheral blood to examine the dynamic and rapidly changing molecular events in the endometrium at this time. Until an accurate, sensitive and reliable and yet non-invasive test is available (which does not disturb the embryo and the implantation process), it is not possible to overcome the ethical dilemma posed on the study of human implantation.

## Supporting information

S1 Table Demographic characteristics in women with recurrent reproductive failure or fertile controls. C: fertile subjects, RIF: recurrent implantation failures, RM: recurrent miscarriages, NS: not significant, SD: standard deviation.
(DOCX)

S2 Table KEGG pathways and GO terms involved in comparison of blood transcriptome between recurrent implantation failures and recurrent miscarriages. (XLSX)
