# Supplementary material for: Transcriptomic profiles in peripheral blood between women with unexplained recurrent implantation failure and recurrent miscarriage and the correlation with endometrium: A pilot study
Source: PLoS One. 2017 Dec 7;12(12):e0189159. doi: 10.1371/journal.pone.0189159 (PMC5720758; doi:10.1371/journal.pone.0189159)
Supplement: S1 Table — C: fertile subjects, RIF: recurrent implantation failures, RM: recurrent miscarriages, NS: not significant, SD: standard deviation. (DOCX) [file pone.0189159.s001.docx]

Supplementary Table 1 Demographic characteristics in women with recurrent reproductive failure or fertile controls

|  | RIF | RM | C |
| --- | --- | --- | --- |
| Age (y), mean±SD | 35.7±2.1 | 37.3±1.9 | 29.0±4.0 |
| Body mass index (kg/m^2^) | 20.8±2.1 | 21.2±2.0 | 20.1±2.3 |
| Cycle length (d), mean±SD | 29.3±3.0 | 29.7±2.2 | 30.0±2.0 |
| Endometrium thickness on the biopsy day, mean±SD | 11.0±4.3 | 10.8±3.7 | 10.5±4.4 |
| Average times of live birth | 0 | 0 | 1.7 |
| Average times of miscarriage | 0.3 | 3.7 | 0 |
| Average times of implantation failure | 5 | 0 | 0 |
|  |  |  |  |

C: fertile subjects, RIF: recurrent implantation failures, RM: recurrent miscarriages, NS: not significant, SD: standard deviation.
